# Changes in Liver Mechanical Properties and Water Diffusivity During Normal Pregnancy Are Driven by Cellular Hypertrophy

**DOI:** 10.3389/fphys.2020.605205

**Published:** 2020-11-23

**Authors:** Karolina Garczyńska, Heiko Tzschätzsch, Anja A. Kühl, Anna Sophie Morr, Ledia Lilaj, Akvile Häckel, Eyk Schellenberger, Nikolaus Berndt, Hermann-Georg Holzhütter, Jürgen Braun, Ingolf Sack, Jing Guo

**Affiliations:** ^1^Department of Radiology, Charité – Universitätsmedizin Berlin, Corporate Member of Freie Universität Berlin, Humboldt-Universität zu Berlin, and Berlin Institute of Health, Berlin, Germany; ^2^iPATH.Berlin Core Unit, Charitá – Universitätsmedizin Berlin, Corporate Member of Freie Universität Berlin, Humboldt-Universität zu Berlin, and Berlin Institute of Health, Berlin, Germany; ^3^Institute for Imaging Science and Computational Modelling in Cardiovascular Medicine, Charité – Universitätsmedizin Berlin, Corporate Member of Freie Universität Berlin, Humboldt-Universität zu Berlin, and Berlin Institute of Health, Berlin, Germany; ^4^Computational Systems Biochemistry Group, Institute of Biochemistry, Charité – Universitätsmedizin Berlin, Corporate Member of Freie Universität Berlin, Humboldt-Universität zu Berlin, and Berlin Institute of Health, Berlin, Germany; ^5^Institute of Medical Informatics, Charité – Universitätsmedizin Berlin, Corporate Member of Freie Universität Berlin, Humboldt-Universität zu Berlin, and Berlin Institute of Health, Berlin, Germany

**Keywords:** liver stiffness, viscosity, pregnancy, hypertrophy, magnetic resonance elastography (MRE), diffusion weighted imaging (DWI), water diffusion, hepatomegaly

## Abstract

During pregnancy, the body’s hyperestrogenic state alters hepatic metabolism and synthesis. While biochemical changes related to liver function during normal pregnancy are well understood, pregnancy-associated alterations in biophysical properties of the liver remain elusive. In this study, we investigated 26 *ex vivo* fresh liver specimens harvested from pregnant and non-pregnant rats by diffusion-weighted imaging (DWI) and magnetic resonance elastography (MRE) in a 0.5-Tesla compact magnetic resonance imaging (MRI) scanner. Water diffusivity and viscoelastic parameters were compared with histological data and blood markers. We found livers from pregnant rats to have (i) significantly enlarged hepatocytes (26 ± 15%, *p* < 0.001), (ii) increased liver stiffness (12 ± 15%, *p* = 0.012), (iii) decreased viscosity (−23 ± 14%, *p* < 0.001), and (iv) increased water diffusivity (12 ± 11%, *p* < 0.001). In conclusion, increased stiffness and reduced viscosity of the liver during pregnancy are mainly attributable to hepatocyte enlargement. Hypertrophy of liver cells imposes fewer restrictions on intracellular water mobility, resulting in a higher hepatic water diffusion coefficient. Collectively, MRE and DWI have the potential to inform on structural liver changes associated with pregnancy in a clinical context.

## Introduction

Pregnancy is a dynamic process involving a series of maternal physiological changes and adaptations that occur to support fetal growth and development. The changes are driven by maternal and placental hormones (estrogen, progesterone, prolactin, and others; Napso et al., 2018) and require considerable morphological and physiological flexibility of the maternal body, both locally and systemically (Moll, 2001; Baeyens et al., 2016; Soma-Pillay et al., 2016; Napso et al., 2018).

The liver is the largest gland in the human body and plays a central role in metabolism. Hepatocytes participate, inter alia, in glucose, lipid, protein, and peptide metabolism (Pedrycz et al., 2014). As a central metabolic organ, the maternal liver undergoes significant changes induced by higher estrogen levels during normal pregnancy. In early pregnancy, when fetal demands are still relatively low, the maternal body stores energy through increased glucose intake and lipogenesis, as well as glycogen accumulation in hepatocytes to prepare for the higher energy consumption by the developing fetus in late gestation. Therefore, maternal cholesterol, triglyceride, and phospholipid levels are elevated from the second trimester until the end of pregnancy (Bacq, 2000–2013; Lain and Catalano, 2007; Pedrycz et al., 2014; Soma-Pillay et al., 2016; Zhang et al., 2017; Napso et al., 2018). In light of these known physiological changes, adjusted standard reference levels of serum markers have been defined for pregnant women (Abbassi-Ghanavati et al., 2009; Jamjute et al., 2009).

In addition, pregnancy-related adaptation of the maternal liver also involves changes in the organ’s biomechanical properties (Ammon et al., 2018; Stenberg Ribeiro et al., 2019). Elastography can quantify biomechanical properties such as stiffness and viscosity of the liver *in vivo*. Studies of pregnant women using ultrasound-based elastography reported that liver stiffness increased during normal pregnancy and returned to baseline values after delivery (Ammon et al., 2018; Stenberg Ribeiro et al., 2019) where intraabdominal pressure was considered an imported contributor. Additionally, it was shown that changes in liver stiffness related to normal pregnancy differed from those caused by pregnancy-related liver disorders such as pre-eclampsia (Frank Wolf et al., 2016; Ammon et al., 2018) and intrahepatic cholestasis of pregnancy (IHC; Cetin et al., 2017). These results indicate that elastography sensitively detects mechanical changes of the liver during pregnancy. However, due to a lack of histological evidence, the underlying biological causes of altered hepatic stiffness during pregnancy are not fully understood, the authors only hypothesized on a possible association of increased stiffness with altered liver perfusion and intra-abdominal pressure during pregnancy.

Magnetic resonance elastography (MRE), an emerging elastography modality based on magnetic resonance imaging (MRI), can quantify the mechanical properties of soft tissues both *in vivo* and *ex vivo*. A compact MRE modality tailored to *ex vivo* tissue investigation of small cylindrical samples has been introduced recently (Ipek-Ugay et al., 2015; Braun et al., 2018; de Schellenberger et al., 2019; Sauer et al., 2019; Everwien et al., 2020). This compact MRE technique enforces well-controlled (cylindrical) boundary conditions permitting analytical solutions of the inverse problem in MRE by utilizing Bessel function. As a result, compact MRE has been shown to provide consistent values of stiffness and attenuation (shear elasticity and shear viscosity) with little degradation by noise. Moreover, MRE examinations can be combined with determination of other quantitative MRI parameters including water diffusion. In order to correlate pregnancy-related biomechanical changes with underlying biology, we used compact MRE (Ipek-Ugay et al., 2015; Braun et al., 2018; de Schellenberger et al., 2019; Sauer et al., 2019; Everwien et al., 2020) to investigate changes in stiffness and viscosity of *ex vivo* liver samples harvested from pregnant rats. With *ex vivo* samples, we examine mainly the dependency of mechanical properties on the underlying microarchitecture of the liver without influence from blood perfusion, which could be a confounding factor. MRE results were paired with diffusion-weighted imaging (DWI) to investigate the effect of pregnancy on hepatic water diffusivity. The water transport and viscoelasticity quantified by MRE and DWI, respectively, provided complementary information that are sensitive to the microstructure of biological tissues.

In addition, we performed extensive histological and biochemical analyses to elucidate possible structural causes of MRE and DWI parameter changes due to pregnancy. Our MRE results might shed light on clinically relevant biophysical changes of the liver detected by ultrasound elastography in pregnant women, however, one must take into consideration the difference between the two imaging modalities in terms of frequency range and data acquisition technique when comparing data obtained from MRE and ultrasound elastography.

## Materials and Methods

All procedures involving animals were approved by the local authority (Landesamt für Gesundheit und Soziales Berlin, Reg. No. T0280/10, T0212/19) and were performed according to institutional guidelines.

### Sample Preparation

Livers were harvested from young adult female Wistar rats (Forschungseinrichtungen für Experimentelle Medizin, FEM, Berlin, Germany; Janvier Labs, Le Genest-Saint-Isle, France) of two groups: (i) pregnant group (P18; sacrificed on 18th day of gestation), *n* = 13; and (ii) non-pregnant group (NP), *n* = 13. The animals were kept in the same animal facility under standard housing conditions for at least 3 days before imaging.

To harvest the liver, rats were anesthetized with an overdose of isoflurane vapor and then decapitated with a rodent guillotine. A slice of liver (approximately 20 mm in height, 5–8 mm in width) was cut from the left lateral lobe of the fresh liver and transferred directly to the sample tube for MR imaging, while the remaining liver tissue was prepared for histological analysis. The width of the liver slice was trimmed to fit the diameter of the sample tube so that the liver slice can be slid into the tube easily without any compression. In 8 rats of each group, the freshly harvested livers were weighed and the blood collected- approx. 0.8 ml per rat in blood collection tubes containing EDTA and lithium heparin (Sarstedt, Germany)- for laboratory analysis.

### Histology and Immunostaining

After harvesting, fresh liver tissue samples (from 8 rats per group) were fixed in 4 % formaldehyde solution (ROTI^®^ Histofix 4 %, Roth, Karlsruhe, Germany) at room temperature for 24 h. After fixation, the samples were dehydrated for 24 h and embedded in paraffin (ROTI^®^ Plast, Roth, Karlsruhe, Germany). The tissue paraffin blocks were sliced into 2 μm thick sections and finally transferred onto Superfrost/Superfrost Plus slides (R. Langenbrinck GmbH, Emmendingen, Germany). As the mechanical properties quantified by MRE directly associate with microarchitecture of the tissue and the arrangement of the structure elements, especially those in the extra cellular matrix (ECM), we have selected the following staining methods for morphological characterization: hematoxylin and eosin (H&E; Mayer’s Hemalum Solution, Merck, Darmstadt, Germany; Eosin Y solution, Sigma-Aldrich, Darmstadt, Germany) and Elastica van Gieson (Merck, Darmstadt, Germany). As hyperproliferation of hepatocytes has been reported in Bustamante et al. (2010), Dai et al. (2011) for pregnant rats, the liver tissue sections were also immunostained for Ki-67 protein. The primary antibody (clone SolA15, eBioscience^TM^ from Thermo Fisher Scientific; Thermo Fisher Scientific Cat# 14-5698-80, RRID:AB_10853185) was pre-incubated with FabuLight secondary antibody (biotinylated goat anti-rat; Jackson ImmunoResearch). Biotin was detected by streptavidin coupled with alkaline phosphatase (ALP) and RED as chromogen [both Dako REAL^TM^ Detection System, ALP/RED, Rabbit/Mouse (Agilent Technologies)], nuclei were counterstained with hematoxylin (Merck Millipore).

Images of stained sections were taken with a BZ-X800 fluorescence microscope (KEYENCE DEUTSCHLAND GmbH, Neu-Isenburg, Germany). Five high-power fields per animal were analyzed. Hepatocytes and Ki67-positive hepatocytes were counted per field of view (FoV) at 40x magnification in H&E-stained and immunostained sections, respectively, using ImageJ software version 1.52v (Schneider et al., 2012). Histological analysis was performed in a blinded manner.

### Magnetic Resonance Elastography and Diffusion-Weighted Imaging

**FIGURE 1:**
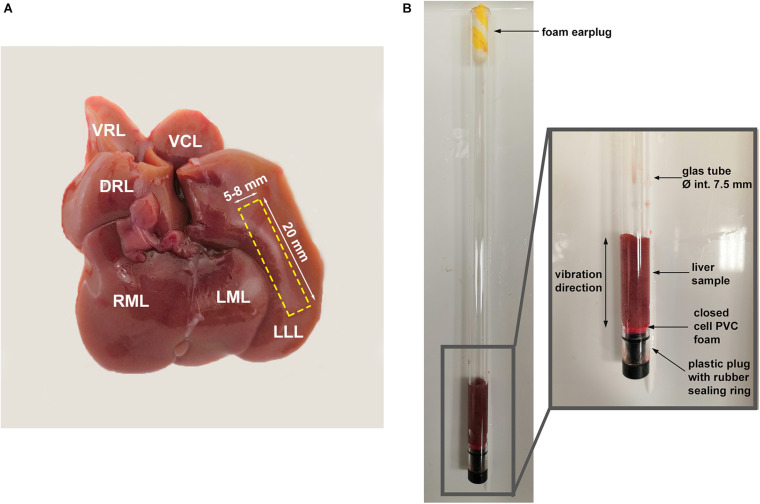
**(A)** Photo of the fresh liver of a non-pregnant rat; rectangular area marked by dashed yellow line indicates the sample taken for MRI examinations. LLL, left lateral lobe; LML, left middle lobe; RML, right middle lobe; DRL, dorsal right lobe; VRL, ventral right lobe; VCL, ventral caudate lobe; and DCL, dorsal caudate lobe. **(B)** Liver sample in the glass tube used for tabletop MRI.

All tissue samples for MRI (P18, *n* = 13; NP, *n* = 13) were taken from the left lateral lobe of the liver which is the largest lobe, facilitating sample preparation (Figure 1A). The MRI measurements started 2 h post-mortem. Liver slices (approximately 20 mm in height, 5–8 mm in width) were cut from the liver. The samples were placed in a glass tube (Figure 1B), which was then inserted in a 0.5-T compact MRI device (Pure Devices GmbH, Würzburg, Germany) for both MRE and DWI.

The compact MRE setup (tabletop MRE) was described in detail in previous publications (Braun et al., 2018; de Schellenberger et al., 2019; Sauer et al., 2019; Everwien et al., 2020). In short, a gradient amplifier (DC 600, Pure Devices GmbH, Würzburg, Germany) and a piezo-actuator (Piezosystem Jena, Jena, Germany) which was directly coupled to the glass sample tube, were integrated into the tabletop MRI device for generating mechanical vibrations and introducing them into the tissue sample. A spin-echo-based MRE sequence as described in Braun et al. (2018) was used for acquiring wave images.

Imaging parameters for tabletop MRE and DWI were similar to those described in Braun et al. (2018), de Schellenberger et al. (2019), Sauer et al. (2019), Everwien et al. (2020). In brief, mechanical vibrations of 800 Hz were excited for MRE acquisition. Dynamic wave propagation was recorded in eight time steps over a wave cycle in one axial 3-mm thick slice with a field of view of 9.6 × 9.6 mm^2^ (64 × 64 matrix size). With a repetition time (TR) of 0.5 s and an echo time (TE) of 20 ms, the total MRE acquisition time was 3 min. DWI was performed with a customized spin-echo sequence (Sauer et al., 2019) using seven *b*-values (50, 175, 300, 425, 550, 675, and 800 s/mm^2^). One 3-mm single slice with an in-plane resolution of 600 μm was acquired with a TR of 1 s and TE of 8 ms, and total acquisition time was 5 min. During one imaging session, MRE and DWI were performed in an interleaved manner, and the MRE/DWI block was repeated five times, resulting in a total acquisition time of 40 min. Sample temperature was kept constant at 30°C for all MRI examinations. During the acquisition time, the liver sample which was sealed in the sample tube and kept at a constant temperature was considered well-conditioned.

For MRE data post-processing, shear wave speed (*c* in m/s) and penetration rate (*a* in m/s) were derived by taking the analytic solution of the fitting of the single profile of complex-valued wave along the *z*-direction based on a Bessel function, as described in Braun et al. (2018), de Schellenberger et al. (2019). *c* represents tissue stiffness while penetration rate *a* is inversely correlated with tissue viscosity. For comparing to results obtained by ultrasound elastography, *c* can be converted to Young’s modulus (*E*) with: *E* = 3*ρc*^2^ where *ρ* is the density, which we assume to be 1 kg/l for all biological soft tissues. For DWI, maps of apparent diffusion coefficient (ADC), which quantifies water diffusivity, were generated with mono-exponential fitting and linear regression analysis taking images at all seven *b*-values. Images with *b*-value of 50 were also used for fitting considering the absence of perfusion in our *ex vivo* samples. Data were post-processed using algorithms written in MATLAB (R2019b, The Mathwork Inc., Natick, MA, United States).

### Statistical Analysis

Mixed analysis of variance (ANOVA) was performed to account for the effects and interactions of two factors present in our data – acquisition time (within-subject factor) and pregnancy (between-subject factor). Normality was tested with both the Shapiro–Wilk test and the Kolmogorov–Smirnov test. Differences between the pregnant and non-pregnant groups were tested using the unpaired *t*-test for normally distributed data and the Mann–Whitney test for datasets that violated the normality assumption. Relationships between data were assessed by Pearson correlation (*n* > 10) and Spearman correlation (*n* < 10). *P*-values below 0.05 were considered statistically significant.

Graphical and statistical analysis was performed with GraphPad Prism (GraphPad Prism 8.01. for Windows, GraphPad Software, San Diego, CA, United States, www.graphpad.com; GraphPad Prism, RRID:SCR_002798) and SPSS 23 (SPSS Inc, Chicago, IL, United States; SPSS, RRID:SCR_002865.

## Results

### General Characterization of Livers From Pregnant Rats

**FIGURE 2:**
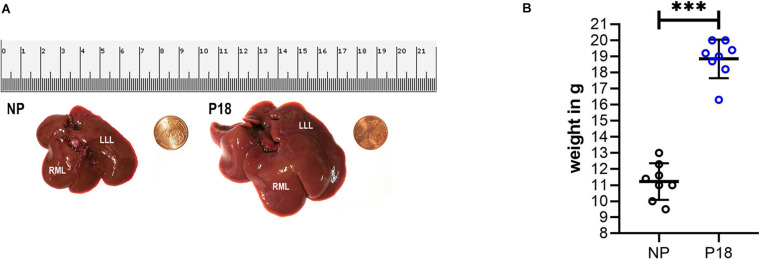
**(A)** Photos of fresh liver from non-pregnant (NP) and pregnant (P18) rats for visual comparison of liver sizes. **(B)** Scatter plots of liver weight in pregnant (P18) and non-pregnant (NP) groups with mean and standard deviation. ***p < 0.001. LLL, left lateral lobe and RML, right middle lobe.

A representative photo of two rat livers is shown in Figure 2A. The liver from a P18 rat was visibly larger compared to that from an NP rat. A significant difference in liver weight was observed between the pregnant and non-pregnant groups (P18: 18.8 ± 1.2 *g vs* NP: 11.2 ± 1.1 *g*, *p* < 0.001, *n* = 8 per group, Figure 2B). The livers from P18 rats were on average 40% heavier than those from non-pregnant rats.

### Histological Evaluation

**FIGURE 3:**
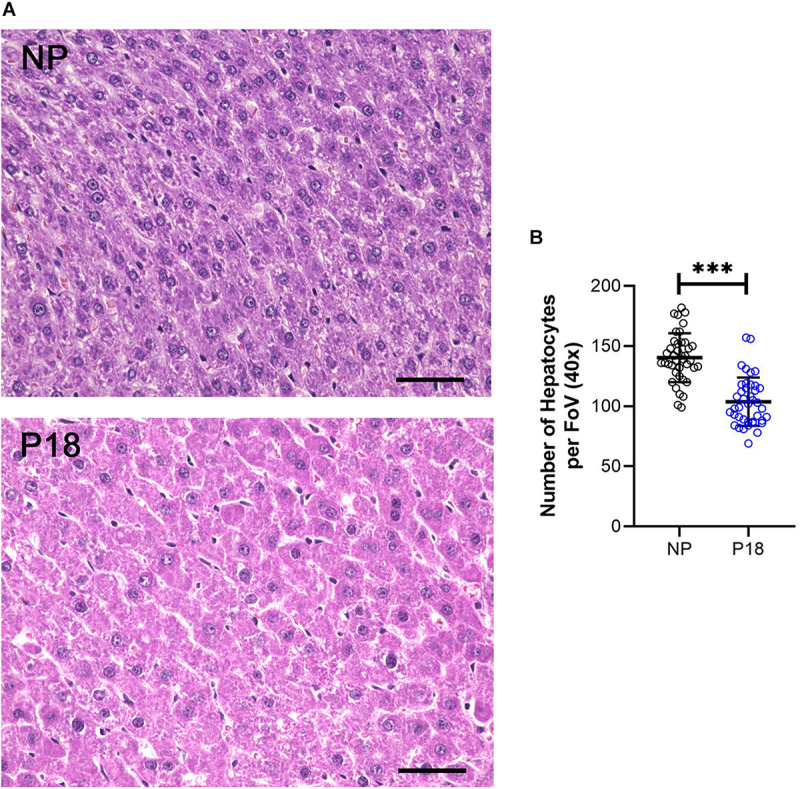
**(A)** Representative H&E-stained liver sections from non-pregnant (NP) and pregnant (P18) rats. Scale bars correspond to 50 μm. Hepatocytes were counted at 40x optical field, five high-power images per animal were analyzed. **(B)** Scatter plots of numbers of hepatocytes per field of view (FoV) in pregnant (P18) and non-pregnant (NP) groups with mean and standard deviation. ^∗∗∗^*p* < 0.001.

The number of hepatocytes per FoV at 40x magnification was significantly lower in the P18 rats than in the NP rats (P18: 104 *vs* NP: 140, *p* < 0.001, *n* = 8 per group), see Figure 3. This 26 % reduction in hepatocyte counts per FoV was characteristic of hepatocyte hypertrophy.

**FIGURE 4:**
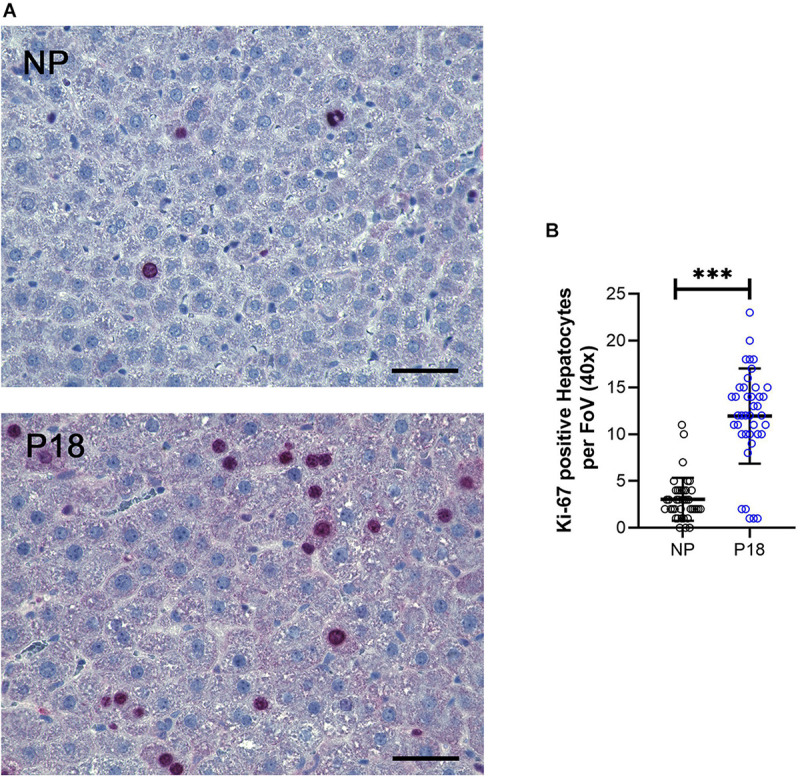
**(A)** Representative Ki-67-immunostained liver sections from non-pregnant (NP) and pregnant (P18) rats. Scale bars correspond to 50 μm. Ki-67-positive hepatocytes were counted at 40x optical field, five high-power images per animal were analyzed. **(B)** Scatter plots of numbers of Ki-67-positive hepatocytes per field of view (FoV) in pregnant (P18) and non-pregnant (NP) groups with mean and standard deviation. ^∗∗∗^*p* < 0.001.

As Ki67 protein is present during all active phases of the cell cycle, it is indicative of cell proliferation. In our samples, we observed a 4-fold increase in Ki67-positive hepatocytes in livers from pregnant rats (P18: 12 *vs* NP: 3, *p* < 0.001, *n* = 8 per group, Figure 4).

**FIGURE 5:**
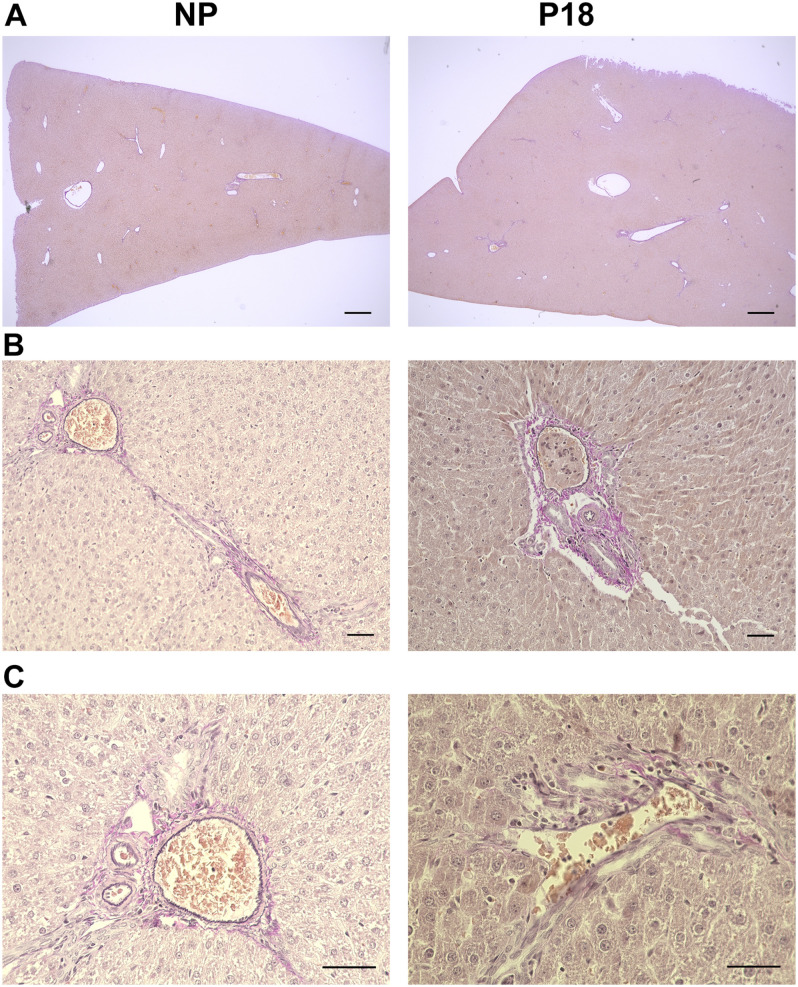
Representative Elastica van Gieson (EvG)-stained liver sections from non-pregnant (NP) and pregnant (P18) rats. **(A)** 2x optical field, scale bars correspond to 500 μm. **(B)** 20x optical field, scale bars correspond to 50 μm. **(C)** 40x optical field, scale bars correspond to 50 μm. Elastic fibers are stained black and collagen red.

Livers from P18 rats did not differ from those of NP rats in terms of hepatic collagen or elastin content, as shown in the H&E- (Figure 3A) and Elastica van Gieson (EvG)-stained slices (Figure 5). Moreover, no visible signs of liver pathologies such as ballooning, steatosis, and inflammation were present in H&E-stained slices in either group.

Additionally, the presence of erythrocytes was visually assessed in both H&E and EvG-stained slices and there was no visible difference between the NP and the P18 groups, as shown in Supplementary Figure 1.

### Biochemical Analysis

A total of 14 serum markers were analyzed. While 4 markers [alanine transaminase (ALT), total protein, creatinine, red blood cells] showed no significant changes during pregnancy, total bilirubin (P18, 2.6 ± 0.3 μmol/l; NP, 2.1 ± 0.7 μmol/l, and *p* = 0.049) and triglyceride (P18, 3.9 ± 1.4 mmol/l; NP, 1.9 ± 0.6 mmol/l, and *p* < 0.001) were significantly increased while the remaining markers showed a significant reduction during pregnancy. The results of biochemical analysis are compiled in Table 1.

**TABLE 1:** Mean serum markers with standard deviation of the pregnant and non-pregnant groups.

Parameter	Pregnant (*n* = 8)	Non-pregnant (*n* = 8)	*p*-value
ALP (U/l)	78.025.0	116.024.2	0.022
AST (U/l)	101.014.2	145.648.6	0.026
ALT (U/l)	69.99.4	79.413.6	0.133
GLDH (U/l)	4.72.4	9.85.5	0.033
Total bilirubin (μmol/l)	2.60.3	2.10.7	0.049
Bile acids (μmol/l)	14.311.8	40.528.8	0.026
Triglyceride (mmol/l)	3.91.4	1.90.6	0.001
Albumin (g/l)	31.01.7	34.32.0	0.003
Total protein (g/l)	58.85.0	61.03.6	0.319
Creatinine (μmol/l)	19.41.4	21.74.1	0.153
Urea (mmol/l)	6.20.6	7.40.9	0.011
Glucose (mmol/l)	5.10.6	7.50.9	<0.001
Hemoglobin (g/l)*	110.03.8	142.54.4	<0.001
Red blood cells (T/l)**	6.00.2	6.72.5	0.106

*ALP, Alkaline phosphatase; AST, Aspartate Transaminase; ALT, Alanine transaminase; and GLDH, Glutamate dehydrogenase.^∗^Pregnant *n* = 7 and non-pregnant *n* = 4; ^∗∗^pregnant *n* = 7 and non-pregnant *n* = 5- the amount of blood was insufficient to execute the test.*

### Magnetic Resonance Elastography

Based on the mixed ANOVA analysis of MRE parameters acquired at multiple time points of the P18 and NP groups, acquisition time, the within-subject factor, had no significant influence on MRE parameters (*c*, *p* = 0.50; *a*, *p* = 0.18) while pregnancy status, the between-subject factor, had a significant effect on both *c* (*p* = 0.01) and *a* (*p* < 0.001). Additionally, there were no significant interactions between these two factors for *c* (*p* = 0.98) or *a* (*p* = 0.44).

**FIGURE 6:**
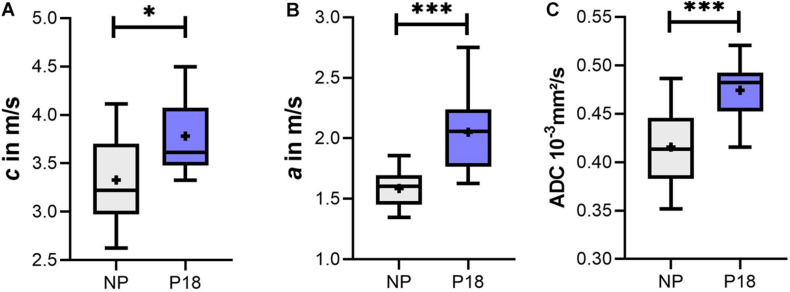
Box plots of **(A)** shear wave speed (*c*), **(B)** penetration rate (*a*), and **(C)** apparent diffusion coefficient (ADC) of the liver in the pregnant (P18) and non-pregnant (NP) rat groups. Data are presented as minimum to maximum with interquartile range and median; + indicates means. ^∗^*p* ≤ 0.05 and ^∗∗∗^*p* ≤ 0.001.

Since acquisition time had no effect on MRE parameters, we averaged both *c* and *a* values of five acquisitions over 40 min for each animal, and compared the difference between the P18 and NP groups using the *t*-test. As shown in Figure 6A, *c* of the P18 group was significantly higher than that of the NP group (P18: 3.8 ± 0.4 m/s *vs* NP: 3.3 ± 0.5 m/s, *p* = 0.012). Similarly, a significant increase in penetration rate *a* was observed in the P18 group compared to the NP group (P18: 2.1 ± 0.3 m/s *vs* NP: 1.6 ± 0.2 m/s, *p* < 0.001, Figure 6B).

**FIGURE 7:**
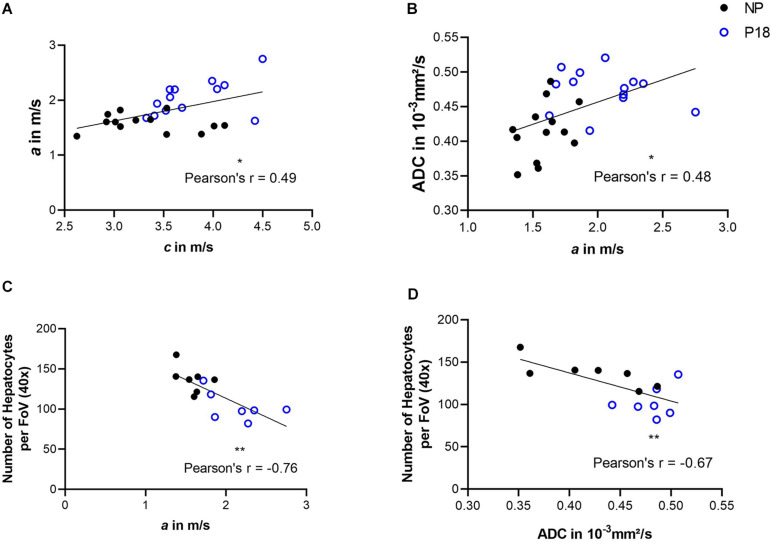
Correlations between: **(A)** shear wave speed (*c*) and penetration rate (*a*); **(B)** *a* and apparent diffusion coefficient (ADC); **(C)** *a* and number of hepatocytes per field of view (FoV); and **(D)** ADC and number of hepatocytes per FoV. Solid black circles represent data from the non-pregnant group (NP) while open blue circles represent data from the pregnant group (P18). ^∗^*p* ≤ 0.05 and ^∗∗^*p* ≤ 0.01.

Correlation analysis was performed by pooling the data from the P18 and NP groups. We observed a positive correlation between *c* and *a* (Pearson’s *r* = 0.49; *p* = 0.011) and a negative correlation between *a* and the number of hepatocytes per FoV (Pearson’s *r* = -0.76; *p* = 0.002), as shown in Figures 7A,C.

We also correlated MRE parameters with biochemical results. There were a total of five significant correlations: between *c* and ALP (Pearson’s *r* = -0.7; *p* = 0.003), *a* and ALP (Pearson’s *r* = -0.65; *p* = 0.006), *a* and albumin (Pearson’s *r* = -0.68; *p* = 0.004), *a* and urea (Pearson’s *r* = -0.54; *p* = 0.033), and *a* and glucose (Pearson’s *r* = -0.76; *p* < 0.001).

### Diffusion-Weighted Imaging

Mixed ANOVA analysis of the DWI data acquired at multiple time points in the P18 and NP groups revealed that the effect of acquisition time (within-subjects factor) was not significant (*p* = 0.19) while the pregnancy status (between-subject factor) significantly influenced the ADC (*p* < 0.001). Also, there was no significant interaction between these two factors (*p* = 0.51). Based on the results of mixed ANOVA analysis, we averaged the ADC values of the five acquisitions over 40 min for each animal and compared the P18 and NP groups with the *t*-test. As shown in Figure 6C, ADC values in the P18 group were significantly higher than in the NP group (P18: 0.47*e*^–3^ ± 0.03*e*^–3^ mm^2^/s; NP: 0.42*e*^–3^ ± 0.04*e*^–3^ mm^2^/s, *p* < 0.001).

For correlation analysis, ADC values from the P18 and NP group were pooled. We observed a positive correlation between *a* and ADC (Pearson’s *r* = 0.48; *p* = 0.013) and a negative correlation between ADC and the number of hepatocytes per FoV (Pearson’s *r* = -0.67; *p* = 0.009). Results of correlation analysis are shown in Figures 7B,D.

We also tested correlation between ADC values and biochemical parameters. Four biochemical parameters – bile acid (Pearson’s *r* = -0.66, *p* = 0.005), triglyceride (Pearson’s *r* = 0.5; *p* = 0.047), albumin (Pearson’s *r* = -0.65; *p* = 0.007), and glucose (Pearson’s *r* = -0.65; *p* = 0.006) – were found to be significantly correlated with ADC values.

Group mean values and standard deviations of the aforementioned imaging parameters are presented in Table 2.

**TABLE 2:** Mean imaging parameters obtained by MRE and DWI and mean liver weight of the pregnant and non-pregnant groups.

Parameter	Pregnant (*n* = 13)	Non-pregnant (*n* = 13)	*p*-value
*c* in m/s	3.80.4	3.30.5	0.012
*a* in m/s	2.10.3	1.60.2	<0.001
ADC in mm^2^/s	0.47*e*^−3^ ± 0.03*e*^−3^	0.42*e*^−3^ ± 0.04*e*^−3^	<0.001
Liver weight in g*	18.9 ± 1.2	11.2 ± 1.1	<0.001

*n = 8 in both groups.

## Discussion

In this study, compact MRE and DWI were used to study biophysical changes occurring in the liver during pregnancy. Our results obtained in rat livers reveal that pregnancy increases stiffness and water diffusivity while reducing viscosity. The biophysical changes identified with these two imaging techniques were correlated with and validated by extensive histological and biochemical analysis.

The most obvious change was a pregnancy-related increase in liver size caused by hepatocyte hypertrophy and hyperproliferation. A pregnancy-related increase in liver weight is well documented for animals (Rosenfeld, 1977; Nuwayhid, 1979; Buelke-Sam et al., 1982; Ahokas et al., 1984). Hollister et al. (1987) were the first to report hepatic growth in pregnancy as a result of hepatocyte hypertrophy. This was later confirmed by other studies (Bustamante et al., 2010; Gielchinsky et al., 2010; Milona et al., 2010; Dai et al., 2011) showing hyperproliferation of hepatocytes (Bustamante et al., 2010; Milona et al., 2010; Dai et al., 2011), increased hepatic DNA content, and an altered hepatic gene expression profile (Bustamante et al., 2010; Dai et al., 2011) during pregnancy. An enhanced liver metabolism during pregnancy could lead to the observed liver growth (Pedrycz et al., 2014). Additionally, elevated estrogen levels in pregnancy (Abbassi-Ghanavati et al., 2009) were reported to induce transient hepatocyte proliferation and liver growth (Fisher et al., 1984; Yager et al., 1994). In the current study, considering the absence of ballooning, steatosis and inflammation based on histopathology, the observed hepatocyte hypertrophy was a result of increased DNA content which was physiological during pregnancy as reported in Bustamante and Dai et al. (Bustamante et al., 2010; Dai et al., 2011). Furthermore, as our histological analysis revealed no evidence of altered structural elements such as collagen and elastic fibers, we conclude that pregnancy-related hypertrophy and hyperproliferation of hepatocytes contributed to the overall increase in liver size and weight in our experiments.

As mentioned in the Introduction, biochemical changes occurring in maternal livers during pregnancy are well studied in humans (Abbassi-Ghanavati et al., 2009; Jamjute et al., 2009; Cunningham, 2010). With the notion that the liver anatomy defers between rats and humans with rats’ liver consisting of four main lobes (Kogure et al., 1999), we compared the biochemical changes in rat maternal liver to that of humans. Most of the changes in serum markers we observed in rat livers were in accordance with results obtained in pregnant women (Abbassi-Ghanavati et al., 2009; Jamjute et al., 2009). Similar to humans, pregnant rats showed an increase in hepatic triglycerides with a concomitant reduction in glucose, which is attributable to the high energy demands during pregnancy. Additionally, hemodilution due to a larger volume of circulating plasma leads to lower albumin levels during pregnancy, a phenomenon observed in both rats (De Rijk et al., 2002; Liberati et al., 2004) and humans (Moll, 2001; Carlin and Alfirevic, 2008; Pedrycz et al., 2014). Nevertheless, there were three main differences between our results and findings known from human studies: firstly, the ALP level decreased significantly in pregnant rats, whereas pregnant women may have up to 3 times higher amounts of ALP compared to non-pregnant women. This difference is due to the fact that ALP produced by rat placenta does not enter maternal serum whereas placental ALP in humans contributes to overall maternal ALP (Boles et al., 1972; Pedrycz et al., 2014). Secondly, in contrast to pregnant women, whose bile acid level is normally elevated (Abbassi-Ghanavati et al., 2009; Jamjute et al., 2009; Pedrycz et al., 2014; McIlvride et al., 2017), rats showed reduced bile acids during pregnancy, which was due to hemodilution as reported in Zhu et al. (2013). Thirdly, unlike humans whose bilirubin level is slightly reduced during pregnancy (Abbassi-Ghanavati et al., 2009; Jamjute et al., 2009), we observed an increase in total bilirubin concentration in the pregnant rats which is accordance with previously published data obtained from Wistar rats (Liberati et al., 2004).

Firstly, liver stiffness of rats measured *ex vivo* in our study is higher than that obtained *in vivo*, as reported in Piecha et al. (2016), Elshaarawy et al. (2020). Aside from the difference between *ex vivo* and *in vivo* conditions and the technical dissimilarity between the two imaging modalities, the frequency used in the current study (800 Hz) was higher than that of Fibroscan used in Piecha et al. (2016), Elshaarawy et al. (2020), 573 Hz, leading to the higher stiffness values. Secondly, pregnancy-related increase in liver stiffness was observed *in vivo* by Ammon et al. (2018) and Stenberg Ribeiro et al. (2019). These authors discussed that elevated liver stiffness might be associated with increased blood flow to the liver and elevated portal pressure. In Ammon et al. (Ammon et al., 2018), the author also proposed a possible association between liver congestion and liver stiffness, however, judging by both the macroscopic liver appearance and the microscopic histologic features, we didn’t observe signs of liver congestion in our samples. More importantly, as the *in vivo* factors such as blood flow and pressure were not present in our *ex vivo* study, we can exclude blood-flow related influences on our data and attribute the stiffness changes that we observed mainly to structural alterations. The expansion of hepatocytes exert force on the cell membrane which lead to elevated intra-cellular pressure and increased mechanical resistance. The observed increase in stiffness reflects the collective behavior of these enlarged cells and is the macroscopic manifestation of elevated intracellular pressure. The expansion and the proliferation of the hepatocytes in the pregnant liver could also reduce intercellular space which lead to decreased friction as reflected by the wave penetration rate (*a*). Thus, in our study, the liver of the pregnant rats appeared more solid-like with lower viscosity. Based on our histology results, there were no other pregnancy-related extracellular matrix alterations such as changes in collagen or elastin fiber content which potentially also influence the mechanical properties of the liver (Feng et al., 2016; Hudert et al., 2018; Heucke et al., 2019). In additional to the aforementioned structural elements that contribute to the mechanical properties of the liver, production of macromolecule such as glycogen which was reported to increase especially at the begin of the pregnancy might also influence the observed hepatic viscoelasticity. We suspect that the accumulation of the multibranched polysaccharide with linear chains could alter the microarchitecture of the hepatocytes, thereby changing the macroscopic viscoelasticity of the liver. However, our study couldn’t provide further detailed insights regarding macromolecule production. Overall, based on our results, we concluded that hypertrophy and hyperproliferation of hepatocytes are the main contributors to the observed changes in hepatic mechanical properties during pregnancy.

Pregnancy-related changes seen in MRE were accompanied by marked increases in ADC values probably due to hypertrophy of the hepatocytes. As cell density per unit area decreases, there are fewer cell membranes, which act as barriers that restrict water mobility within the hepatocytes, and water diffusion increases. This is consistent with data (Kele and van der Jagt, 2010) showing that reduced cellularity (number of cells per area) due to cell hypertrophy increases water diffusivity. Although the hyperproliferation of hepatocytes potentially reduces water diffusivity (Le Bihan, 2013), we assume that – in light of the considerably expanded liver volumes in our group of pregnant rats, effects of hypertrophy dominated over hyperproliferation in our ADC values.

The inverse correlations between the number of hepatocytes per FoV and imaging parameters *a* and ADC confirms liver hypertrophy to be the main contributor to both the pregnancy-related reduction of viscosity and increase of water diffusion.

Despite encouraging results, our study has limitations. Firstly, biochemical and histological examinations were performed only in a subgroup of rats. Secondly, we only imaged a small portion of the left lateral hepatic lobe, while clinical *in vivo* MRI normally covers the whole liver. However, as normal pregnancy usually affects the whole maternal liver, we do not expect significant regional difference. Finally, as we investigated only *ex vivo* liver samples, possible confounders for changes of the liver stiffness during pregnancy observed *in vivo* such as intra-abdominal pressure and blood perfusion (Millonig et al., 2010; Mueller, 2016; Piecha et al., 2016; Ammon et al., 2018) were not considered. To assess the influence of these factors, *in vivo* studies using animal models are warranted.

In conclusion, using a compact tabletop MRI scanner, we observed increased stiffness and water diffusion accompanied by decreased viscosity in *ex vivo* rat liver specimens obtained from rats with normal pregnancy. Our results suggest that these changes in biophysical properties were mainly caused by pregnancy-related hypertrophy and hyperproliferation of hepatocytes as supported by biochemical and histological examinations. Finally, the maternal liver during pregnancy mechanically transforms from a soft-viscous to a more solid-rigid state. MRE and DWI have the potential to inform on structural changes of the maternal liver in a clinical context.

## Data Availability Statement

The raw data supporting the conclusions of this article will be made available by the authors, without undue reservation.

## Ethics Statement

The animal study was reviewed and approved by Landesamt für Gesundheit und Soziales Berlin.

## Author Contributions

KG: data acquisition, investigation, formal analysis, data curation, and writing – original draft. HT and LL: software and validation. AK: histological investigation and writing – review and editing. A-SM: investigation and writing – review and editing. AH, ES, NB, and H-GH: writing – review and editing. JB: funding acquisition, resources, methodology, project administration, supervision, and writing – review and editing. IS: conceptualization, funding acquisition, resources, methodology, project administration, supervision, and critical revision of manuscript. JG: conceptualization, data curation, formal analysis, funding acquisition, investigation, methodology, visualization, supervision, writing – original draft, and critical revision of manuscript. All authors fully qualify for authorship and have approved the final version of the manuscript.

## Conflict of Interest

The authors declare that the research was conducted in the absence of any commercial or financial relationships that could be construed as a potential conflict of interest.

## Supplementary Material

Supplementary Figure 1 Selected **(A)** H&E-stained and **(B)** EvG-stained liver sections from non-pregnant (NP) and pregnant (P18) rats. Scale bars correspond to 500 μm. Visually, similar amount (both high and low) of erythrocytes were found in NP and P18, indicating that the number of erythrocytes is not uniquely different in the pregnant rats compared with the pregnant ones.
