# Supplementary figures and images for: Changes in Liver Mechanical Properties and Water Diffusivity During Normal Pregnancy Are Driven by Cellular Hypertrophy
Source: Front Physiol. 2020 Nov 23;11:605205. doi: 10.3389/fphys.2020.605205 (PMC7719759; doi:10.3389/fphys.2020.605205)

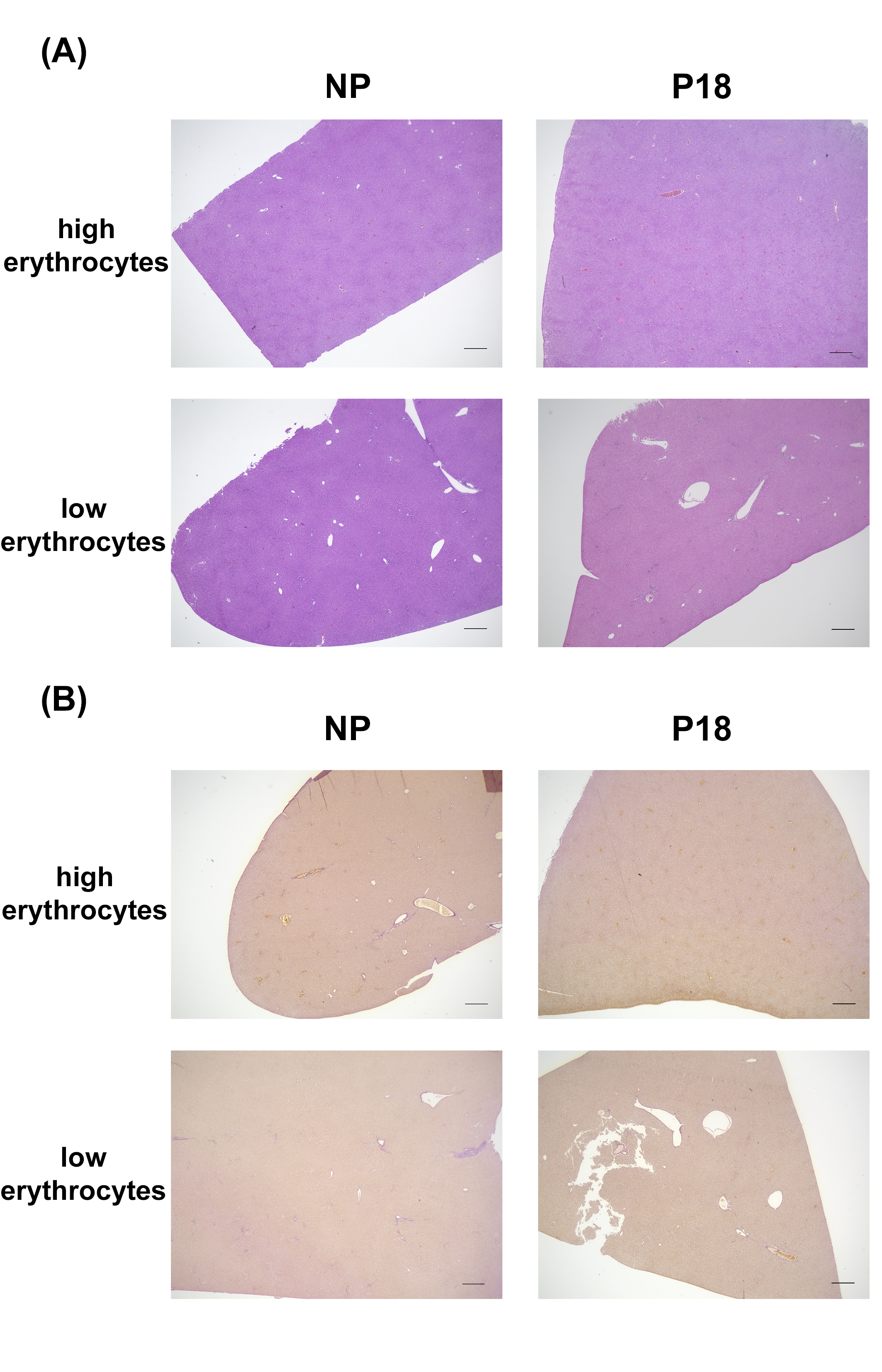

Supplement: Supplementary Figure 1 — Selected (A) H&E-stained and (B) EvG-stained liver sections from non-pregnant (NP) and pregnant (P18) rats. Scale bars correspond to 500 μm. Visually, similar amount (both high and low) of erythrocytes were found in NP and P18, indicating that the number of erythrocytes is not uniquely different in the pregnant rats compared with the pregnant ones. [file Image_1.TIF]
